# The Emerging Role of Insulin Receptor Isoforms in Thyroid Cancer: Clinical Implications and New Perspectives

**DOI:** 10.3390/ijms19123814

**Published:** 2018-11-30

**Authors:** Veronica Vella, Roberta Malaguarnera

**Affiliations:** 1 School of Human and Social Sciences, “Kore” University of Enna, 94100 Enna, Italy; vellave@hotmail.com; 2 Endocrinology, Department of Clinical and Experimental Medicine, University of Catania, Garibaldi-Nesima Hospital, 95122 Catania, Italy; 3 Endocrinology, Department of Health Sciences, University Magna Graecia of Catanzaro, 88100 Catanzaro, Italy

**Keywords:** insulin receptor isoforms, thyroid cancer, insulin/IGF system, IR-A/IGF2 autocrine loop, hybrids receptors, thyroid cancer stem cells

## Abstract

Thyroid cancer (TC) is the most common endocrine tumor. Although the majority of TCs show good prognoses, a minor proportion are aggressive and refractory to conventional therapies. So far, the molecular mechanisms underlying TC pathogenesis are incompletely understood. Evidence suggests that TC cells and their precursors are responsive to insulin and insulin-like growth factors (IGFs), and often overexpress receptors for insulin (IR) and IGF-1 (IGF-1R). IR exists in two isoforms, namely IR-A and IR-B. The first binds insulin and IGF-2, unlike IR-B, which only binds insulin. IR-A is preferentially expressed in prenatal life and contributes to development through IGF-2 action. Aggressive TC overexpresses IR-A, IGF-2, and IGF-1R. The over-activation of IR-A/IGF-2 loop in TC is associated with stem-like features and refractoriness to some targeted therapies. Importantly, both IR isoforms crosstalk with IGF-1R, giving rise to the formation of hybrids receptors (HR-A or HR-B). Other interactions have been demonstrated with other molecules such as the non-integrin collagen receptor, discoidin domain receptor 1 (DDR1), and the receptor for the hepatocyte growth factor (HGF), Met. These functional networks provide mechanisms for IR signaling diversification, which may also exert a role in TC stem cell biology, thereby contributing to TC initiation and progression. This review focuses on the molecular mechanisms by which deregulated IR isoforms and their crosstalk with other molecules and signaling pathways in TC cells and their precursors may contribute to thyroid carcinogenesis, progression, and resistance to conventional treatments. We also highlight how targeting these alterations starting from TC progenitors cells may represent new therapeutic strategies to improve the clinical management of advanced TCs.

## 1. Introduction

Thyroid cancer (TC) is the tumor with the fastest increasing incidence in the western world [1,2,3]. The increased TC risk is attributable not only to an improvement in thyroid-based diagnostic procedures, but also to some rising risks factors, such as insulin resistance conditions [4], therapies [5,6,7], and environmental carcinogens [8], which are responsible for molecular alterations that are specific to TC [9].

The increasing incidence involves mainly well-differentiated TC histotypes, which are considered to be low risk tumors, because patients outcome is excellent, with a 5-year disease specific survival of >90% [10]. Appropriate thyroidectomy, followed or not by adjuvant radioactive iodine (RAI) therapy and active surveillance, is the common treatment against these tumor histological variants and associated distant metastases [10]. However, roughly two thirds of patients become RAI-resistant [11]. Rare, poorly-differentiated thyroid carcinoma (PDTC) and anaplastic thyroid cancer (ATC) are often unresponsive to RAI treatment and, therefore, highly aggressive. For all TC subtypes that have lost RAI uptake ability, nowadays, optimal clinical management is still lacking, even though new perspectives are appearing [12]. Therefore, there is an urgent need for therapies that can slow down the progression of these aggressive tumors. 

Evidence from the literature suggests that TCs resistant to RAI are dependent on the activation of specific signaling pathways for the maintenance of their malignant phenotype [13]. Thus, these tumors may only be responsive to therapies targeting the molecular signals important for their growth and survival. Although thyroid function and proliferation are primarily regulated by the thyroid-stimulating hormone (TSH), other pathways such as mitogen-activated protein kinase (MAPK), phosphoinositide 3-kinase (PI3-K), mammalian target of rapamycin (mTOR), and the insulin growth factors (IGF) system play an equally important role for the proliferation and growth of thyrocytes as well as their precursors/stem cells. Recently, genetic or epigenetic alterations preventing the normal process of self-renewal, proliferation, and differentiation of these progenitor cells have been considered as the possible origin of thyroid malignant transformations (4). The best studied and already identified molecular alterations in TC include conditional or constitutive deregulation of MAPK/PI3-K/mTOR/IGF cascades.

Activation of the MAPK cascade via mutations and/or rearrangements of genes REarranged during Transfection (RET), Rat sarcoma (RAS), and proto-oncogene B-Raf (BRAF) occurs in ~70% of well-differentiated TCs. Among these abnormalities, BRAF is the most frequently mutated gene in TC, and a predictor of poor clinical prognosis and recurrence [14]. TC also shows mutations in PI3-K signaling effectors, such as Phosphatase and tensin homolog (PTEN) and phosphatidylinositol-4,5-bisphosphate 3-kinase catalytic subunit alpha (PIK3CA) [15]. The PI3-K pathway may also be over-activated in TC by non-mutational mechanisms, such as dysregulation of the IGF system. In fact, we have previously demonstrated that both IGFs are locally produced in TCs: IGF-1 by stromal cells and IGF-2 by malignant thyrocytes, with higher values in malignant tissues compared to normal tissue [16]. In addition, IGF-1R and IR have been found to be overexpressed, especially in PDTCs. As a consequence of this overexpression, IR/IGF-1R hybrid formation and increased IGF-1 response occur. Another important finding is that overexpressed IR is predominantly present as the fetal IR-A isoform, and that malignant thyrocytes acquire the ability to produce IGF-2. This autocrine loop involving IGF-2 and the isoform IR-A is activated in different cancers and particularly in the PDTC histotypes [16,17,18], and exerts a crucial role in regulating stem-like properties, metastases, and resistance to therapies [19,20,21,22]. Indeed, IR-A, IGF-1R, and their ligands IGF-1 and IGF-2 are overexpressed in human thyroid progenitor cells, with a prevalence in progenitors from TC cells, where IGF-2 exerts a role in stimulating self-renewal and thyrosphere volume [20]. Furthermore, besides the formation of IR/IGF-1R hybrid receptors, the functional crosstalk between IR-A and other tyrosine kinase receptors such as the non-integrin collagen receptor, discoidin domain receptor 1 (DDR1), the receptor for the Hepatocyte Growth Factor (HGF) Met, and other still unknown partners might add more complexity to the contribution of IR-A in TC pathogenesis. 

Based on these experimental observations indicating that deregulation of survival pathways and tyrosine kinase receptors-dependent signaling may play an important role in TC development and progression, multikinase inhibitors (MKI) (i.e., sorafenib and lenvatinib) have been approved for use in treatment [11,23]. These MKI have been shown to enhance RAI uptake via sodium iodine symporter (NIS) upregulation [24,25]. However, so far, the real efficacy of these drugs, showing also an antiangiogenic activity, is uncertain. Indeed, they can improve the progression free survival, but not the overall survival [23]. Therefore, new therapeutic options are needed for aggressive TC subtypes.

Given the well-recognized role of IR-A/IGF-2 overexpression/activation in thyroid malignant transformation, TC de-differentiation, and stem-like properties, this pathway could represent a new target for TCs that are unresponsive to RAI therapy, or to re-differentiation therapy using MKI [23,26]. However, a selective inhibition of IR-A is hard to reach, given the very little difference in structure between IR-A and the “metabolic“ isoform IR-B. Likely, the identification of new molecular partners of IR-A, recruited after insulin and/or IGF-2 stimulation, could open new and valuable therapeutic perspectives.

Here, we highlight the role of IR isoforms, especially IR-A and its molecular partners, in TC stem cell biology, and as consequence, in TC development, progression, and resistance to conventional treatments. Targeting the overactivated IR-A/IGF-2 loop could sensitize stem-like cells to classical therapies and reduce relapse rates for the most aggressive TC histotypes.

## 2. Thyroid Tumorigenesis

### 2.1. Onset of TC

Cancer of the thyroid gland is the most common malignancy of endocrine organs and the fastest increasing tumor worldwide [1]. More than 95% of thyroid tumors originate from the neoplastic transformation of the thyroid follicular epithelium, and include a heterogeneous group of neoplasms with specific clinical, pathological, and molecular features. Well-differentiated carcinomas (including papillary and follicular histotypes) and poorly-differentiated and undifferentiated tumors (including anaplastic cancer) are the three main histological subgroups of TCs. Well-differentiated TC is the most frequent and indolent type, representing more than 80% of all thyroid tumors, with a mean 5-year overall survival rate of 90% [2]. By contrast, undifferentiated or ATCs are very rare and are the most advanced and aggressive histotypes [27].

The pathological molecular mechanisms explaining the origin of TC and the different behavior between the different subtypes are currently only partially understood. The evidence in tumor cell biology and genetics has provided some hypotheses to explain the onset of thyroid oncogenesis. The classical multistep model assumes a stepwise process of de-differentiation [28]. According to this hypothesis, normal differentiated mature thyrocytes give rise to papillary or follicular, and finally, to most undifferentiated ATCs, by accumulating multiple somatic mutations in genes such as RAS, RET, Neurotrophic tropomyosin receptor kinase (NTRK), Gs-protein (GSP), TSH-Receptor (TSH-R) and alterations of tumor suppressor p53 family members [29,30,31,32,33,34]. In this stochastic model, TC initiation is monoclonal, and tumor heterogeneity is acquired by multistep mutations that deregulate cell proliferation, differentiation, and survival processes [28].

The recently proposed alternative hypothesis relies on the presence, within the thyroid gland, of cells with fetal/stem-like features [35]. Based on this “cancer stem cell (CSC) carcinogenesis” model, undifferentiated and pluripotent cells, which under normal conditions represent a source for regenerating cells, may acquire mutations or epigenetic changes, altering their self-renewal, proliferation, and differentiation abilities. These modifications could give rise to malignant transformation from adult stem cells or their committed progenitors toward specific TC stem cells, which in turn become the origin of cancer cells [36,37,38]. This model postulates a hierarchy of thyroid precursors cells as the potential cause of distinct TC histotypes, and is supported by the observation that several signaling pathways involved in adult stem cells biology (i.e., the Wingless–type Mouse mammary tumor virus integration site family member (Wnt) pathway, Hedgehog (Hh) and Notch signaling pathways) are also deregulated in TCs. Furthermore, there are close relationships between gene expression profile detected in main components of the solid cell nests (such as oncofetal fibronectin, p63, Carcinoembryonic antigen (CEA), and Cytokeratin 34 beta E12 (CK34bE12) and some thyroid malignancies, suggesting that such undifferentiated remnant embryonic stem cells may be the niche for thyroid cell survival, and may play an important stem cell-like role in the origin of several thyroid disorders, including TC [39,40].

### 2.2. Molecular Abnormalities and Deregulated Signaling Pathways Involved in Thyroid Oncogenesis

Recent advances in our understanding of the molecular alterations responsible for thyroid carcinogenesis provide compelling evidence for the crucial role of mutational or non mutational abnormalities in components of survival-signaling cascades, such as TSH-R, MAPK, PI3-K/AKT, mTOR, and the IGF pathways [41] (see Table 1). 

#### 2.2.1. TSH-R

TSH has long been recognized as the major stimulus for thyrocytes function, differentiation, and proliferation [41]. TSH induces thyrocyte growth directly by binding to its own receptor, and indirectly by stimulating the autocrine and/or paracrine production of other growth factors such as Vascular Endothelial Growth Factor (VEGF) [42] and amyloid precursors [43]. Additionally, insulin, IGF-1, and serum factors are essential for TSH-mediated thyrocytes proliferation [41,44,45]. The crucial role of TSH-R and its downstream signaling in TC onset and evolution comes from large epidemiological studies showing a strong association between serum TSH levels and increased TC incidence in patients with thyroid nodules [46,47]. Furthermore, in vivo experiments in mice harboring BRAF mutation have shown that TSH cooperates with oncogenic BRAF to induce thyroid tumorigenesis, partially via cyclic adenosine monophosphate (cAMP) [48]. The exact mechanisms responsible for TSH contribution to BRAF-induced transformation are largely unknown. The TSH-R engagement induces the activation of protein kinase A (PKA) via cAMP, and consequent cell cycle progression and cell proliferation in response to growth factors, especially IGF-1 or insulin [41,49]. A variety of targets have been associated with the mitogenic output of TSH-dependent signaling. These include PKA dependent and independent signals, as well as other proliferation pathways such as MAPK, PI3-K/AKT/mTOR, Wnt/β-catenin, and the IGF system. This well-regulated signaling network and the heterogeneity of TSH targets further amplifies and diversifies the final response of TSH-dependent pathway [49,50].

#### 2.2.2. MAPK

The most common molecular abnormalities responsible for papillary TC (PTCs) initiation and progression include chromosomal alterations generating chimeric oncogenes or point mutations in proto-oncogenes such as Ret, NTRK, RAS, and BRAF [51,52]. Each of these events is linked to specific TC etiologic factors and to specific histotypes. In particular, rearrangements of RET gene (RET/PTC) (especially RET/PTC1 and RET/PTC3) occur in 5 to 30% of PTCs, and have been associated with DNA fragility consequent to radiation exposure [53]. RAS mutations are uncommon, except in the follicular variant of PTCs, which also show Paired-box gene 8/Peroxisome Proliferator-Activated Receptor gamma (PAX-8/PPARγ) translocations. The most common mutated gene is BRAF, which appears activated in 35–60% of PTCs [53]. The origin of point mutations in BRAF and RAS remain largely unknown. BRAF mutations, as well as alterations in the p53 gene, have often been found in ATCs, suggesting that these molecular abnormalities may be involved in TC progression to poorly-differentiated and aggressive phenotypes [53]. Moreover, constitutive activation of the MAPK pathway may be responsible for TC resistance to MKI [54]. Common features of all these molecular abnormalities along the MAPK pathway are that they do not overlap in the same tumor, and are involved in signaling from the different growth factors and membrane receptors that are responsible for TC formation, maintenance, and progression. However, in a significant proportion of TC cases, alterations involving MAPK cascade are absent, suggesting that other signaling pathways may play a role in thyroid carcinogenesis. 

#### 2.2.3. PI3-K/AKT/PTEN

Enhanced signaling through the PI3-K/AKT pathway has been recognized as a common feature of thyroid follicular adenoma and carcinoma. PI3-K is activated in thyrocytes by several growth factors such as insulin/IGF-1, Epidermal Growth Factor (EGF), Hepatocyte Growth Factor (HGF) [41]. Furthermore, PI3-K is essential for thyrocytes proliferation under TSH stimulation [45,55,56]. Roughly 40% of well-differentiated TCs and more than 50% of highly aggressive TCs show PTEN inactivation consequent to downregulation or gene silencing [57]. Point mutations or copy number changes of PIK3CA and Protein Kinase B (PKB also known as AKT) are found in ~23% of ATCs, sometimes together with either RAS or BRAF mutations [58]. AKT activation may be required to inhibit apoptosis in TC, and is likely present in tumors harboring RAS mutations. AKT activation is more evident in the invasive regions of TC and in lymph nodes or distant metastases [58]. Furthermore, it has been observed that PI3-K/AKT deregulation leads to upregulation of Wnt/β-catenin pathway, which induces or promotes retention of the de-differentiated status commonly observed in advanced TCs [59].

#### 2.2.4. mTOR/p70S6K

Among the crucial shared downstream effectors of both MAPK and PI3-K pathways, the kinase mTOR has directly been associated with a hyperproliferative phenotype [60,61]. Human PTCs show increased levels of p-p70S6K and ribosomal protein S6 (rpS6), the two major mTOR targets. Highly phosphorylated p70S6K and rpS6 exert a potential role in thyroid proliferation and activity downstream TSH-R by increasing the protein levels of cyclins D1 and D3 [62,63]. These mTOR downstream effectors are required for the mitogenic response triggered by TSH/cAMP and PI3-K on thyroid follicles both in vivo and in vitro [55,62]. Compared to normal thyrocytes, TC cells are unresponsive for growth to TSH, forskolin, or cAMP [64] and do not require PKA for mTOR activity [65]. Many TCs may have modified the upstream control of mTOR activity from TSH/cAMP-PKA to either RAS-RAF-MAPK and/or PI3-K/AKT [65]. Indeed, most human TC cell lines showing mutations in effectors of MAPK and/or PI3-K pathways, are cAMP/TSH independent and unresponsive to PKA control. This is in line with the observation that patients affected by advanced TC entirely lose TSH dependence on tumor growth. However, targeting only MAPK and/or PI3-K is often insufficient to completely block mTOR activity. Therefore, a combination therapy using mTOR inhibitors with drugs that directly target the driver-oncoprotein opens promising doors for the treatment of advanced and the most aggressive TCs.

#### 2.2.5. Insulin/IGF System

Another signaling pathway contributing to generating proliferative responses in both normal and tumor thyrocytes is the insulin/IGF axis [41,45]. 

In vivo evidence shows that in physiology, the stimulation of thyroid growth by TSH is partially dependent on other growth hormones including insulin and/or IGF-1 [66]. Indeed, both hormones in concert with cAMP modulate the expression of Thyroid Transcription Factor-2 (TTF-2), which mediates the transcription of thyroid-specific genes such as Thyroglobulin (Tg), Thyroperoxidase (TPO), and TSH-R [67].

The crosstalk between TSH and the insulin/IGF axis appears to also play a role in abnormal thyroid cell proliferation. In fact, the pro-tumorigenic role of TSH is irrelevant in the absence of growth factors, but it is greatly potentiated by the presence of insulin and/or IGF-I [68,69]. Multiple molecular alterations in the IGF system make more efficacious the pro-tumorigenic actions of TSH. An early event in thyroid carcinogenesis [16,70] is represented by the overexpression of IGF-1, IGF-1R, IGF-2, and IR in TC cells, which is responsible for cellular transformation, proliferation, and apoptosis suppression [71]. In TC, IR is predominantly expressed as the “pro-mitogenic” isoform A (IR-A), which binds—with high affinity—to not only insulin, but also to IGF-2, which is produced by cancer cells in autocrine manner. The activation of the IGF-2/IR-A loop has a recognized role in tumor progression, de-differentiation, and resistance to therapies. Indeed, the overexpression of IR-A is a feature of poorly-differentiated anaplastic or stem-like TC cells [16,20]. Conversely, IGF-1R expression decreases with cancer de-differentiation [72], suggesting that the IGF-2/IR-A loop exerts a more important role than the IGF-1/IGF-1R loop in thyroid cells de-differentiation, stemness, tumor progression, and metastasis [73]. 

Furthermore, the coexisting presence of IGF-1R and IR in many tumors including TCs causes the formation of IR/IGF-1R hybrids receptors [74]. Both IR isoforms can equally combine with IGF-1R, leading to the formation of hybrids containing IR-A (HR-A) or IR-B (HR-B). HRs, likely HR-A as consequence of the overexpression of this isoform in TCs, have been found both in well-differentiated and in poorly-differentiated/undifferentiated PTCs [18,75]. In these tumors, the presence of HRs has important consequences for cancer responses to both insulin and IGFs [18,74]. (see also Section 3.2).

Another mechanism through which the IGF system regulates TC development, growth, proliferation, invasion, and biological behavior, thereby contributing to thyroid tumorigenesis, is the collaboration with other pro-mitogenic signaling pathways such as MAPK, PI3-K, Janus kinase/signal transducer, and the activator of transcription (JAK/STAT) cascades [70].

In the following paragraphs, we will pay particular attention to the role of IR isoforms, especially IR-A, and their interaction with other signaling pathways in TC initiation, progression, and maintenance.

## 3. Crosstalk between IR Isoforms with Other Signaling Pathways and Molecules in TCs

**Figure 1:**
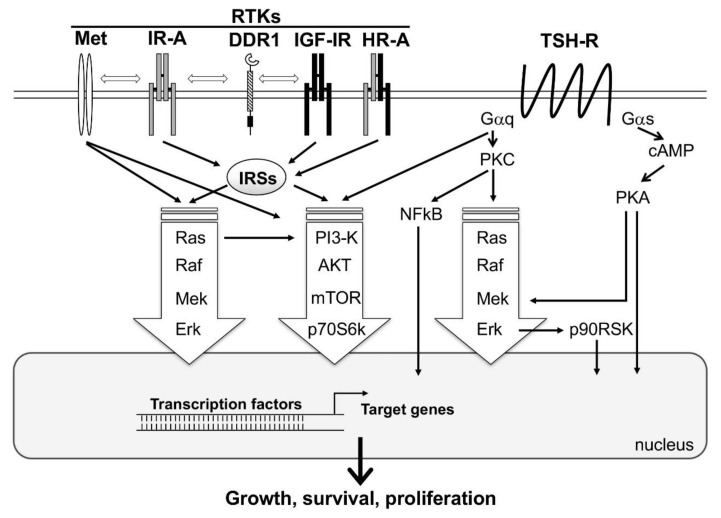
A simplified representation showing the interplay between the major signaling cascades activated by tyrosine kinase receptors (RTKs) and TSH-Receptor (TSH-R). Activation of TSH-R on the cell surface by TSH results in the activation of two major classes of G proteins, namely Gαq and Gαs. Gαs, through the second messenger, cAMP, activates the major signaling pathway PKA/Mek/Erk. In addition, PKA may directly stimulate transcription factors in the nucleus, resulting in the activation of target genes involved in thyroid follicular cells function, growth, and survival. Gαq activates the signaling cascades PKC/Ras/Raf/ERK/p90RSK, PI3-K/AKT/mTOR/p70S6k, and PKC/NFkB. RTKs (i.e., Met, IR-A, DDR1, IGF-1R and HR-A) directly or via IRSs, induce MAPK (Ras/Raf/Mek/Erk) and PI3-K/AKT/mTOR/p70S6k signaling cascades. All these pathways interact with each other, resulting in a multitude of cross-talks. MAPK and PI3-K cascades are common to both TSH-R and RTKs downstream-mediated signaling, and are represented with big arrows, as they are the major players of follicular cell proliferation.

It is now well accepted that insulin/IR-mediated signaling relies on heterogeneous molecular networks composed of various membrane molecules and transmembrane and matrix receptors. This crosstalk contributes to modulating IR isoforms physiological functions and, when deregulated, may play an important role in pathological conditions such as diabetes, insulin-resistance, and cancer [44,72,76]. This complicated signaling network could play an important role also in thyroid tumorigenesis (Figure 1).

### 3.1. IR-A/DDR1 Crosstalk

Among IR-A molecular partners, we have identified the non-integrin collagen-binding tyrosine-kinase receptor DDR1. DDR1 is involved in cell adhesion and proliferation. Usually, it is expressed in tumors with invasive phenotypes, suggesting a possible role in tumor progression and metastasis [77]. The first evidence for functional crosstalk between IR signaling and DDR1 comes from a Stable Isotope Labeling by/with Amino acids in Cell culture (SILAC) proteomic analysis conducted in mouse fibroblasts overexpressing only IR-A [78]. This study identified DDR1 as a novel IR-A molecular substrate, preferentially recruited after IR-A binding to IGF-2 compared to insulin [78]. Thereafter, in human breast cancer cells, we confirmed that DDR1 is a signaling partner of IR-A [79]. Indeed, DDR1 was able to physically associate and co-localize with IR-A after insulin or IGF-2 exposure. In turn, exposure to IGFs induced DDR1 protein up-regulation through a PI3-K/AKT/miR199-5p circuit which was able to modulate IGFs biological responses [80]. DDR1 specific silencing reduced breast cancer cell proliferation, migration, and the activation of IR downstream signaling. Furthermore, DDR1 knock-down markedly inhibited IR protein and mRNA expression through both transcriptional and post-transcriptional mechanisms, while DDR1 overexpression elicited the opposite effects. Similar results were obtained in mouse fibroblasts lacking IGF-1R and transfected with IR-A and DDR1 [81], in SKUT 1 myosarcoma cells [81], and in TC cells [82]. All these findings suggest that this functional crosstalk between IR signaling and DDR1 could be a general mechanism contributing to IR signaling diversification in several types of cancers, including TC [79].

Therefore, in TC cells or precursors overexpressing IR-A and showing over-activated IR-A/IGF2 loops, the functional crosstalk between IR-A and DDR1 may be important in modulating the biological response to insulin and IGF2, or to regulate stem-like features. Interestingly, the inhibition of this network may potentially provide a new opportunity to selectively inhibit unwanted mitogenic IR-A mediated effects without affecting IR-B metabolic functions.

### 3.2. IR/IGF-1R Interaction

The existence of physical and functional crosstalk between IR isoforms and the homolog receptor IGF-1R was proven by the observation that in cells and tissues overexpressing both receptors, such as breast tumors and TCs, IR may heterodimerize with IGF-1R leading to the formation of HRs. HRs behave more similarly to IGF-1R than to IR as they show a high binding affinity for IGF-1 but not for insulin [83]. Both IR-A and IR-B isoforms may randomly combine with IGF-1R (HR-A and HR-B) with the same efficiency [74]. Therefore, the content of hybrids containing IR-A or IR-B correlates to their relative expression. In TCs, especially poorly-differentiated histotypes and in TC progenitor cells, where IGF-1R is overexpressed and IR is mainly present as isoform A, it is reasonable to expect a high content of HR-A. This latter receptor binds with high affinity to IGF-1 and IGF-2, and with lower affinity to insulin, in contrast to HR-B, which behaves as a selective receptor for IGF-1 [74]. These differences in ligand availability and binding capacities confer to each HR different functional features, and may give rise to different biological responses. Indeed, HR-A, but not HR-B, mediates post-receptor signaling and biological effects, such as migration and cell proliferation, stimulated by both insulin and IGFs. Furthermore, the hybrid containing IR-A, and not IR-B, exclusively activates the IGF-1R phosphorylation cascade and IGF-1R-mediated biological functions also after insulin binding [74]. Therefore, the signaling pathway mediated by HRs is unique and differentially activated in a tissue-specific manner and in particular pathological conditions such as hyperinsulinemia. In summary, in TC, the increased expression of IR-A and IGF-1R represents a mechanism to over-activate the biological effects stimulated by insulin, IGF-1, and IGF-2, to induce diversification of the signaling mediated by IR-A homodimers, and to favor the crosstalk between IR-A and IGF-1R, thereby giving a selective growth advantage to TC cells.

### 3.3. IR Crosstalk with Met

The oncogene Met is a trans-membrane tyrosine kinase receptor with a high affinity for the HGF. Met is overexpressed and activated in several human malignancies including PTCs, where it has been found to be deregulated in approximately 70% of tumors, and has been proposed as a negative prognostic factor [84,85]. In PTCs, Met may regulate HGF-mediated biological effects such as survival, morphogenesis, angiogenesis, cell adhesion, and invasive growth. Met may also affect the tumor microenvironment by modulating inflammatory cells, tumor-associated fibroblasts, blood vessels, and extracellular matrices (ECMs) [86]. 

A cooperation between IR and Met was originally demonstrated in hepatocytes, where IR/Met interaction involves preferentially IR-B and controls liver glucose metabolism [87]. However, IR/Met cooperation has been also described in several human malignancies, including PTCs [88,89,90], which overexpress Met, IR (preferentially as isoform A), and the corresponding ligands. In these cancers, it is possible that the crosstalk between IR and Met not only leads to the physical formation of IR/Met hybrid receptors, but also induces the simultaneous activation of both IR and Met-downstream signaling pathways involved in cell motility, growth, and morphogenesis [72]. So far, no studies have been performed to investigate whether both IR isoforms are equally able to combine with Met. However, a growing body of evidence suggests that the involvement of one or the other IR isoforms in the crosstalk with Met is dependent on IR isoform expression levels in a given tissue rather than on IR isoforms specific properties [87,88]. For example, in TCs, which overexpress IR-A, it is possible to suppose that the hybrid formation with Met involves preferentially this “more mitogenic” IR variant. Therefore, it is possible that in this tumor, IR-A/Met hybrids, amplifying the biological response to HGF and insulin/IGFs, may contribute to cancer progression, chemotaxis, and haptotaxis [86]. Interestingly, an important relationship between the Met and AKT pathway has been described in TC stem cells derived from aggressive histotypes, where an increased phosphorylation status of both Met and AKT confer motile and metastatic features [91]. These findings further support a role for targeting Met/IR signaling interactions as a valid therapeutic option to treat invasive and aggressive TC. However, further studies are necessary to better clarify the biological significance and implications of IRA-Met interaction in TC.

## 4. Role of Insulin and IRA/IGF2 Loop in TC Cells and Stem Cells Biology

As previously mentioned, it has already been demonstrated that human TCs often overexpress IR-A [16,19,70]. In TC cells, IR-A may be activated by the autocrine production of IGF-2. Alternatively, the cancer-associated stromal cells, located in the tumor microenvironment, can produce IGF-2, thus activating IR-A in a paracrine way. The activated IGF-2/IR-A loop has been associated with tumor aggressiveness, loss of differentiation [70], and the acquisition of a stem-like phenotype [20]. Indeed, TC cells under specific culture conditions in vitro may form thyrospheres enriched with stem/progenitor cells, which are strongly positive for several stem cell markers such as Octamer-binding transcription factor-4 (Oct-4), ATP-binding cassette subfamily G member 2 (ABCG2), CD-133, CD-44, and Homeobox protein NANOG (Nanog). Thyrospheres also have the ability to self-renew, giving rise to a new generation of spheres. In addition, under proper conditions, they can re-differentiate into cells with strong expression of the specific thyroid differentiation markers such as Tg, TPO, TSH-R, Thyroid Transcription Factor-1 (TTF-1), and PAX-8. Compared to thyrospheres obtained from normal thyroid tissue, cancer thyrospheres express higher levels of stemness markers and almost absent levels of thyroid differentiation markers. In addition, thyrospheres derived from PDTCs exhibit high levels of IR/IGF-1R, in terms of both transcripts and proteins, and have a high relative expression of IR-A. In particular, we found that IR-A relative expression ranged 65–86% in cancer thyrospheres, 50–65% in normal thyrospheres, and 40–45% in normal thyroid primary cultures or differentiated sphere-derived thyrocytes. Moreover, poorly-differentiated cancer thyrospheres produce a high amount of both IGF-1 and IGF-2. This latter growth factor has a role in cancer progenitors’ biology due to its ability to activate high expression of IR-A and IGF-1R. Specifically, IGF-2 stimulates the self-renewal properties of the thyroid precursors, thus favoring cancer thyrosphere expansion. Interestingly, the IGF-2 production decreases under cell differentiation culture conditions [20].

The IGF system analysis of cancer thyrospheres is further complicated by the possibility of the generation of IR/IGF-1R hybrids by random assembly IR and IGF-1R hemidimers [72,92,93]. As a consequence, hybrids formed by IR-A dimers are expected to have a higher affinity for IGF-2, thus further increasing the tumor progression abilities of the TC progenitors. 

**Figure 2:**
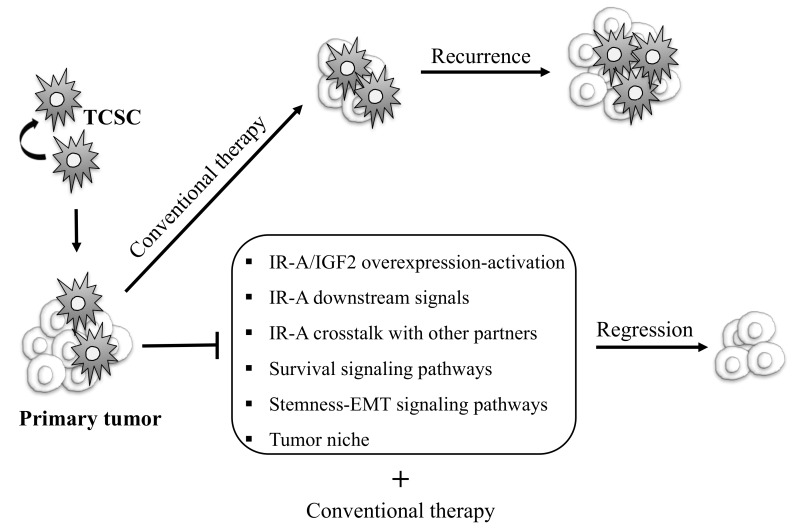
Schematic representation of possible therapeutic strategies targeting thyroid cancer initiating progenitor cells to eradicate TC. Thyroid cancer stem cells (TCSC) are considered to be responsible of TC initiation, progression, therapeutic resistance, and recurrence. Conventional therapy fails to destroy these cells, contributing to tumor relapse and metastases. TCSC-focused therapies may include strategies aiming to inhibit IR-A/IGF2 overexpression and activation, IR-A-mediated mitogenic signals, the crosstalk between IR-A and other molecular partners, signaling pathways regulating survival, stemness, EMT programs, and the tumor niche. This strategy combined with conventional therapy could allow us to eradicate tumor cells and obtain complete remission.

Overall, these data suggest that the IR-A/IGF2 pathway plays a critical role in self-renewal and proliferation of TC stem/progenitor cells, and that a high IR-A:IR-B ratio is a feature of stemness, while a decrease in IR-A:IR-B ratio is associated with thyroid cell differentiation. In support of these data, we have previously found that, in PDTCs, the elevated IR/IGF-1R expression and the high IR-A/IR-B ratio concur with the over-activation of the IR-A/IGF2 loop, and are associated with the acquisition of stem-like features, a more invasive phenotype, and de-differentiation [82]. So far, the molecular mechanisms by which IR-A overexpression/activation in TC are associated with cell stemness and de-differentiation remain poorly understood. Moreover, the means by which the two IR isoforms may differently regulate thyroid cell differentiation is also unclear, although it is likely that the two isoforms activate different intracellular signaling, regulating EMT and stemness, and recruiting different molecular partners [94]. These considerations have some implications. Indeed, targeting IR-A/IGF-2 activation starting from the TC precursor cells may represent a viable option for inducing a re-differentiating program in advanced and RAI-resistant TCs. However, to better potentiate conventional therapy and obtain tumor regression, we likely need at the same time to target other effectors of the network connected to IR signaling, such as those regulating the EMT process, cell stemness, and the tumor niche (Figure 2).

## 5. Clinical Implications and New Perspectives

Several lines of evidence support the observation that in the last few years, TC incidence has increased across all countries [1,2,3]. Although most cases of well-differentiated TC show a good prognosis, two thirds of them become resistant to current RAI therapy. RAI resistance is also a feature of ATC, which is the most aggressive TC variant.

Therefore, new therapeutic options are needed for these TC subtypes. MKI has been proposed and approved as a new re-differentiating therapy for aggressive TCs, but so far, the results have been unsatisfactory. In this scenario, combined therapies could open new perspectives for the clinical management of advanced and RAI-refractory TCs. Based on preclinical data, the insulin/IGF system is often deregulated in PDTCs and in TC stem-like precursors, which usually show increased expression of IR-A, IGF-1R, and IGF-2, and as a consequence, increased formation of hybrid HR-A and over-activation of IR-A/IGF-2 autocrine loop. The activation of this circuit is often associated with the loss of differentiation, stem-like features, tumor progression, and resistance to oncologic therapies [20]. In light of these considerations, future anti-TC strategies aiming to block IGF axis overactivation and its interactions with several components of the pathways involved in stemness and EMT programs will need now to be considered as potential new approaches to safely targeting IR-A activation and inducing a re-differentiating program in TC, without interfering with IR-B metabolic function.

Furthermore, another concept to be highlighted is that hyperinsulinemia, present in insulin-resistant conditions, may favor IR-A/IGF-2 loop activation, thereby potentiating IR-A-dependent mitogenic functions via MAPK/PI3-K/mTOR signaling activation, and worsening the prognosis of TC [4]. Yet, hyperinsulinemia by increasing the bioavailability of IGF-1 and IGF-2 may favor tumor progression and the activation of IGF-1R, IR/IGF-1R hybrids, IR-A, and their interaction with other molecular partners [79,81,95].

Based on this observation, it is reasonable to expect that healthy diet, physical activity, insulin sensitizers (i.e., metformin and PPAR-γ agonists), or inhibitors of SGLT2, could exert beneficial effects on the prevention and treatment of TC developed in insulin-resistant patients [96]. In support of this hypothesis is the observation that metformin inhibits the growth, volume, and size of the thyroid nodule [97,98,99]. Moreover, metformin reduces the effects of insulin on the growth and formation of thyrospheres obtained from undifferentiated TC cells, and potentiates the anticancer effects of doxorubicin and cisplatin [96]. However, so far, studies aimed at evaluating the efficacy of all these therapeutic options as add-on therapies for patients with TC in the context of insulin-resistance are lacking.

## Figures and Tables

**Table 1:** Molecular abnormalities and deregulated signaling pathways involved in TC.

Signaling Pathway	Research Findings	References
**TSH-R**	TSH stimulates the production of other growth factors (VEGF, amyloid precursors)	[42,43]
	TSH/cAMP cooperates with insulin and IGF-1 to regulate thyroid cell proliferation, cell cycle progression and the expression of Tg, TTF-1 and TSH-R mRNA levels	[41,45,49]
	Serum TSH levels directly correlate to TC incidence in patients with thyroid nodules	[46,47]
	TSH cooperates with oncogenic BRAF to induce thyroid tumorigenesis, partially via cAMP	[48]
**MAPK**	MAPK is activated as a consequence of aberrant expression of proto-oncogenes such as Ret, NTRK, RAS and BRAF	[51,52]
	RET/PTC rearrangement is present in 5–30% of PTCs	[53]
	Ras mutations and PAX-8/PPAR-γ translocation are present in PTCs follicular variant	[53]
	BRAF is mutated in 35–60% of PTCs	[53]
	BRAF mutation and p53 alterations have been found in ATCs	[53]
**PI3-K/AKT/PTEN**	Several growth factors such as insulin/IGF-1, EGF, HGF activate PI3-K/AKT/PTEN signaling pathway	[41]
	PI3-K/AKT/PTEN signaling is essential for thyrocytes proliferation under TSH stimulation	[55,56]
	PTEN inactivation is present in about 40% of PDTCs and more than 50% of highly aggressive TCs	[57]
	Point mutations or copy number changes of PIK3CA and AKT1 have been found in ~23% of ATCs sometimes together with either RAS or BRAF mutations	[58]
	AKT1 activation is more evident in invasive region of TCs, in lymph nodes or distant metastasis	[58]
	PI3-K/AKT deregulation upregulates Wnt/β-catenin pathway, inducing de-differentiation	[59]
**mTOR/p70S6K**	TSH proliferative signaling involves mTOR kinase without activating AKT1	[62]
	mTOR downstream effectors are required for the mitogenic response triggered by TSH/cAMP and PI3-K on thyroid follicles	[55,62]
	Many TCs modify the upstream control of mTOR activity becoming cAMP/TSH independent and unresponsive to PKA control	[65]
**Insulin/IGF system**	Insulin/IGF system contributes to generate proliferative responses mediated by TSH	[41,45,66,68]
	Insulin/IGF system together with cAMP, modulates the expression of TTF-2	[67]
	The overexpression of IGF-1, IGF-1R, IGF-2 and IR in TC cells induces cellular transformation, proliferation and apoptosis suppression	[71]
	Overexpression of IR-A is a feature of poorly differentiated, anaplastic or stem-like TC cells	[16,19]
	IGF-1R expression decreases with cancer de-differentiation	[72]
	IGF-2/IR-A loop exerts a more important role than the IGF-1/IGF-1R loop in thyroid cells de-differentiation, stemness, tumor progression and metastasis	[73]
	HRs (likely HR-A) are present both in well differentiated and in poorly differentiated/undifferentiated PTCs	[18,75]
	HRs affect cancer responses to both insulin and IGFs	[18,74]
	Insulin/IGF system crosstalks with other pro-mitogenic signaling pathways such as MAPK, PI3-K, JAK/STAT cascades	[70]

## Abbreviations

TC	Thyroid Cancer
IGFs	Insulin-like growth factors
IR	Insulin Receptor
IGF-1R	IGF-1 Receptor
HR	Hybrid receptor
HR-A	Hybrid receptor isoform A
HR-B	Hybrid receptor isoform B
DDR1	Discoidin Domain Receptor 1
HGF	Hepatocyte Growth Factor
RAI	Radioactive Iodine
MKI	Multikinases Inhibitors
TSH	Thyroid Stimulating Hormone
TSH-R	Thyroid Stimulating Hormone Receptor
MAPK	Mitogen-Activated Protein Kinase
PI3-K	Phosphoinositide 3-Kinase
mTOR	mammalian Target of Rapamycin
BRAF	proto-oncogene B-Raf
RAS	Rat sarcoma
RET	REarranged during Transfection
PDTCs	Poorly Differentiated Thyroid Carcinomas
ATCs	Anaplastic Thyroid Cancers
PTCs	Papillary Thyroid Cancers
TCSC	Thyroid Cancer Stem Cells
CSC	Cancer Stem Celsl
ECM	Extra-cellular Matrix
EMT	Epithelial-Mesenchymal Transition
PKA	Protein Kinase A
PKC	Protein Kinase C
RTKs	Receptors Tyrosine Kinase
SHC	Src Homology domain C-terminal
Gαs	G-protein (s) subunit alpha
Gaq	G-protein (q) subunit alpha
IRS1	Insulin Receptor Substrate-1
AKT	Protein Kinase B
MAPK	mitogen-activated protein kinase
ERK	extracellular-signal-regulated kinase
p70S6K	p70S6 kinase
p90RSK	p90 ribosomal S6 kinase
NFkb	nuclear factor kappa-B
PTEN	Phosphatase and tensin homolog
TFs	Transcription Factors
NTRK	Neurotrophic tropomyosin receptor kinase
GSP	Gs-Protein
CEA	Carcinoembryonic antigen
CK34bE12	Cytokeratin 34 beta E12
VEGF	Vascular Endothelial Growth Factor
EGF	Epidermal Growth Factor
cAMP	cyclic adenosine monophosphate
TPO	Thyroperoxidase
Tg	Thyroglobulin
TTF-1	Thyroid Transcription Factor-1
TTF-2	Thyroid Transcription Factor-2
PAX-8/PPAR-γ	Paired-box gene 8/Peroxisome Proliferator-Activated Receptor gamma
MEK	Mitogen-activated protein kinase kinase
SILAC	Stable Isotope Labeling by/with Amino acids in Cell culture
P53	Tumor suppressor p53
Wnt	Wingless-type Mouse mammary tumor virus integration site family member
Hh	Hedgehog
JAK	Janus kinase
STAT	Signal Transducer and Activator of Transcription
Oct-4	Octamer-binding transcription factor-4
ABCG2	ATP-binding cassette subfamily G member 2
Nanog	Homeobox protein NANOG
PI3KCA	phosphatidylinositol-4,5-bisphosphate 3-kinase catalytic subunit alpha
